# Rheumatoid meningitis presenting with a stroke-like attack treated with recombinant tissue plasminogen activator: a case presentation

**DOI:** 10.1186/s12883-018-1143-z

**Published:** 2018-09-06

**Authors:** Masashi Akamatsu, Futaba Maki, Hisanao Akiyama, Daisuke Hara, Masashi Hoshino, Yasuhiro Hasegawa

**Affiliations:** Department of Neurology, St. Marianna University School of Medicine, 2-16-1 Sugao, Miyamae, Kawasaki, Kanagawa 216-8511 Japan

**Keywords:** Rheumatoid meningitis, Recombinant tissue plasminogen activator (rt-PA), Stroke mimics

## Abstract

**Background:**

Rheumatoid meningitis presenting with a stroke-like attack (RMSA) is a rare manifestation of rheumatoid arthritis (RA). When the patients arrive within the time-window for recombinant tissue plasminogen activator (rt-PA) infusion therapy, no diagnostic protocol has been established.

**Case presentation:**

A 55-year-old woman was brought by ambulance to our hospital with complaints of sudden-onset dysarthria and left arm numbness. The National Institutes of Health Stroke Scale (NIHSS) score was 5, and the Alberta Stroke Program Early CT Score was 8. She was diagnosed with acute embolic stroke. At 4 h, 6 min after onset, intravenous administration of rt-PA (alteplase, 0.6 mg/kg) was started. Her neurological deficits improved rapidly, and her NIHSS score was 1. Brain MRI was then performed. There was no hemorrhagic transformation, but the MRI findings were not compatible with ischemic stroke. She had a past history of RA diagnosed 6 months earlier, and she had been treated with methotrexate (10 mg daily). She was diagnosed with RMSA, and continuous infusion of methylprednisolone 1000 mg daily was started for 3 days. The high signal intensity on the FLAIR image disappeared.

**Conclusion:**

CT-based decision-making for rt-PA injection is reasonable, but MRI is needed for the early diagnosis of RMSA. In this case, it is particularly important that neither adverse events nor bleeding complications were observed, suggesting the safety of CT-based thrombolytic therapy in RMSA.

## Background

Rheumatoid vasculitis is one of the extra-articular complications of rheumatoid arthritis (RA). Although there are no reports of the exact incidence of central nervous system (CNS) complications of RA [1], they are considered relatively rare [2]. Among such complications, there have been extremely rare cases of sudden-onset focal brain dysfunction that resemble a stroke, which is known as rheumatoid meningitis presenting with a stroke-like attack (RMSA) [3, 4]. The safety of thrombolysis in patients with this stroke mimic remains uncertain. The case of a patient who, 6 months after being diagnosed with RA, was brought to our hospital as an emergency due to the abrupt onset of focal neurologic deficits and was treated with recombinant tissue plasminogen activator (rt-PA) is described.

## Case presentation

**Fig. 1:**
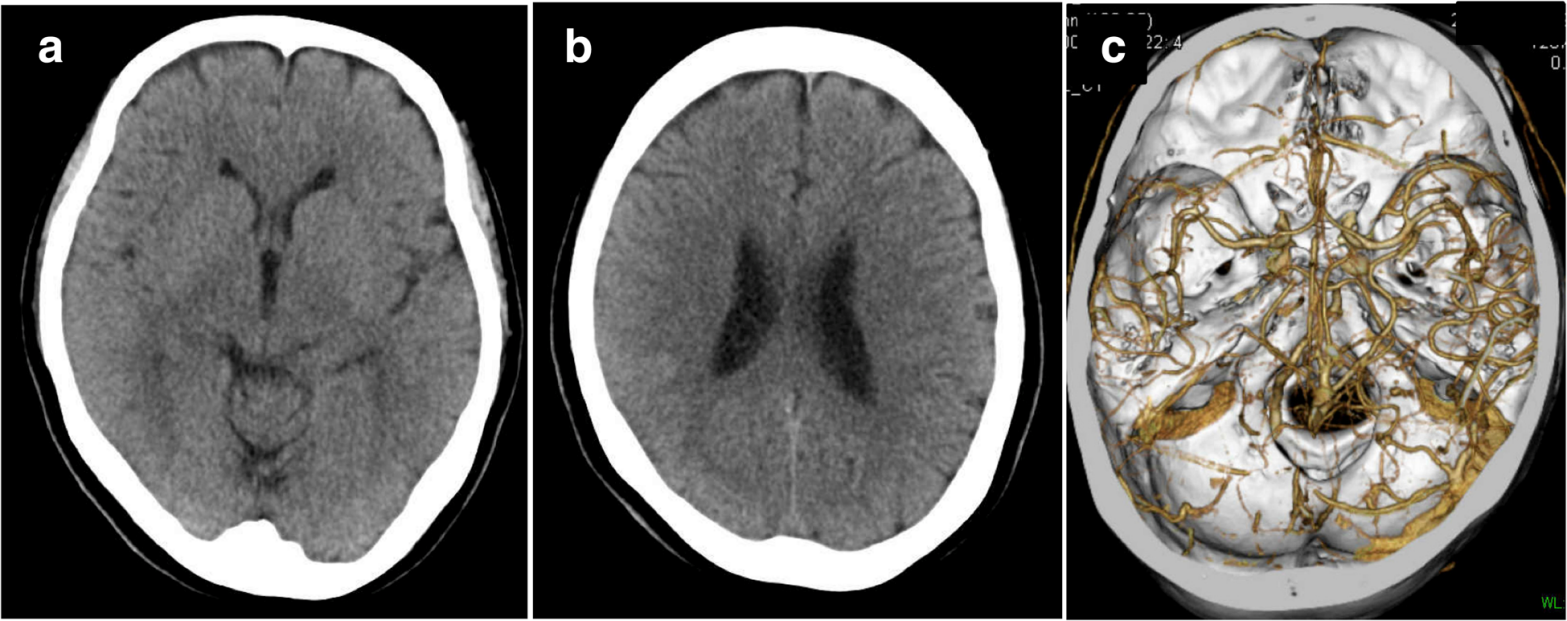
Brain CT images (**a**, **b**) do not show a hypodense area at admission, but equivocal findings of effacement in the right temporoparietal region (ASPECT score, 8). CT angiography (**c**) shows no vascular occlusion or stenosis. CT = computerized tomography, ASPECTS = Alberta Stroke Program Early CT Score

**Fig. 2:**
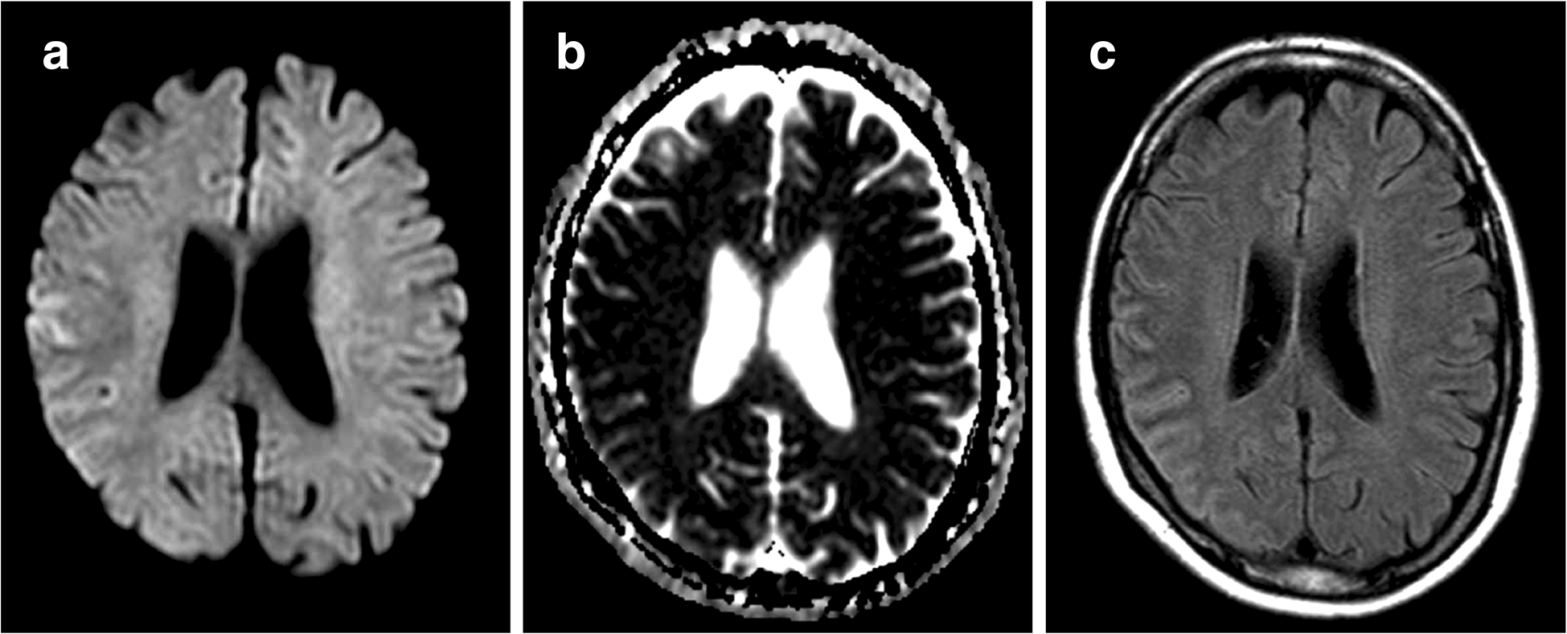
MRI findings at day 1. DWI (**a**) demonstrates a linear high-intensity lesion in the right frontotemporal cortex. Reduced ADC of this lesion is seen on the ADC map (**b**), and this cortical lesion appears as a high-intensity lesion on the FLAIR image (**c**). MRI = magnetic resonance imaging, DWI = diffusion-weighted imaging, ADC = apparent diffusion coefficient, FLAIR = fluid attenuated inversion recovery

A 55-year-old woman suddenly became aware of speech difficulty and left arm numbness at 11:00 pm while talking on the telephone with her daughter. She was taken to our hospital by ambulance. She was diagnosed with RA 6 months earlier, and she had been treated with methotrexate at a dose of 10 mg daily. She had a history of herpes simplex encephalitis from 30 years earlier. Her blood pressure was 155/80 mmHg, pulse rate was 80/min and regular, and temperature was 36.8 °C. ECG was normal. Her Glasgow coma scale was E4 V4 M6, and neurological examination demonstrated dysarthria, left hemiparesis, left-sided sensory impairment, and left unilateral spatial neglect. The National Institutes of Health Stroke Scale (NIHSS) score was 5. Head computed tomography (CT) showed no obvious lesions except effacement of the cortical sulci in the right parietal lobe, and the Alberta Stroke Program Early CT Score (ASPECTS), a 10-point quantitative topographic CT scan score, was 8. No arterial occlusion or stenosis was seen on CT angiography (Fig. 1). Blood tests showed a platelet count of 274 × 10^3^/μl, prothrombin time International Normalized Ratio (PT-INR) of 1.07, and activated partial thromboplastin time (APTT) of 25.6 s (APTT-control 31.0 s). She was diagnosed with acute embolic stroke in the right parietal lobe, and there was no contraindication to intravenous thrombolytic therapy. At 4 h 6 min after onset, intravenous administration of rt-PA was started in accordance with the Japanese guideline (alteplase, 0.6 mg/kg) [5, 6], with an intravenous drip infusion of 30 mg of edaravone, a free radical scavenger, over a period of 30 min. Head magnetic resonance imaging (MRI) was performed at 1 h, 30 min after starting the thrombolytic therapy (Fig. 2). Diffusion-weighted imaging (DWI) demonstrated a linear high-intensity lesion in the right temporoparietal cortex. The lesion was demonstrated to be a hypodense linear lesion on the apparent diffusion coefficient (ADC) map image. This cortical lesion was seen as a high-intensity lesion on fluid-attenuated inversion recovery (FLAIR) imaging. These MRI findings were not compatible with acute ischemic stroke. Her neurological deficits improved rapidly (NIHSS score: 5 on admission, 1 at 24 h after thrombolytic therapy).

**Fig. 3:**
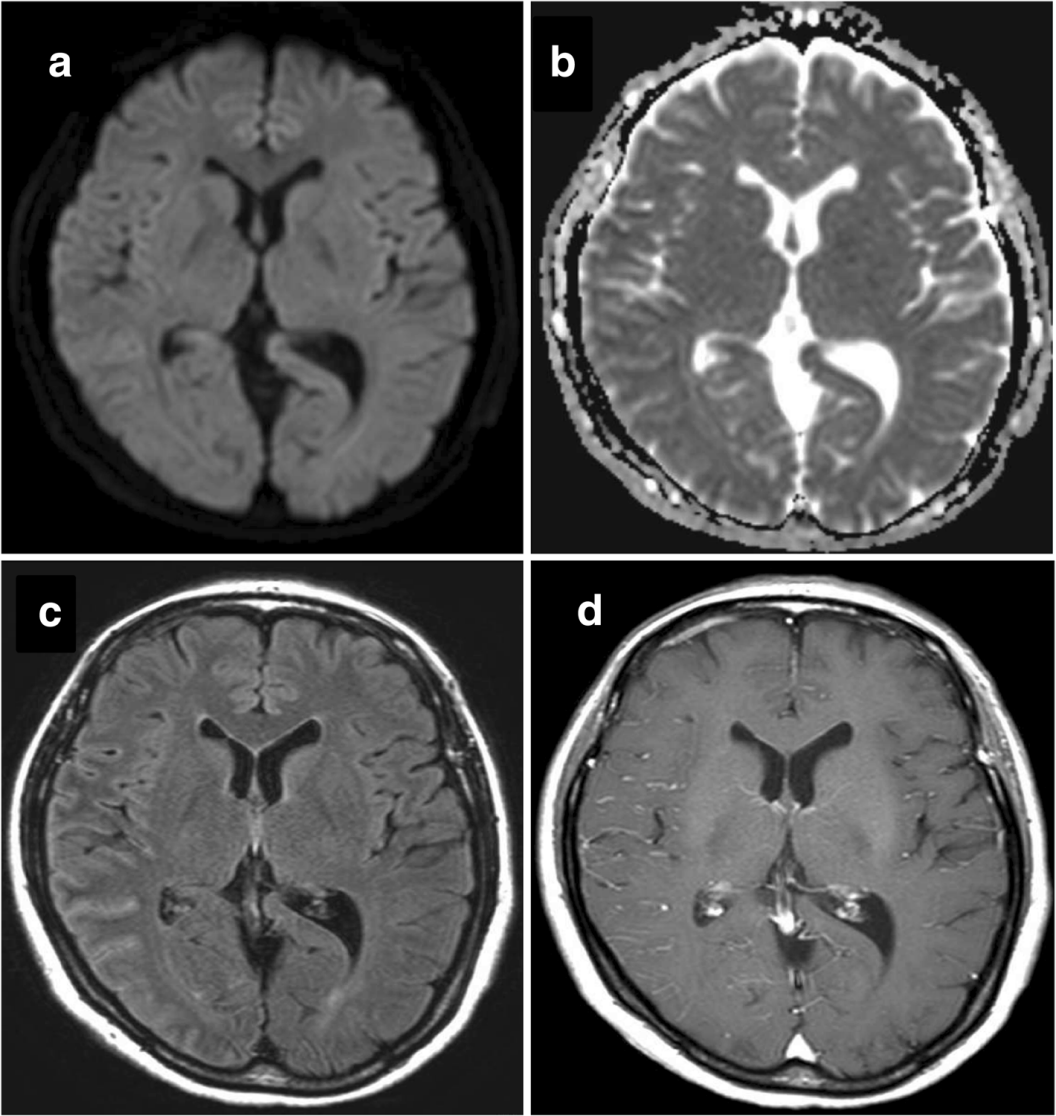
MRI images at day 3. The abnormal findings at day 1 have disappeared on DWI (**a**) and the ADC image (**b**), but the high-intensity area remains on the FLAIR image (**c**) at day 3. There is no definite enhancement of the lesion on gadolinium-enhanced T1-weighted imaging (**d**). MRI = magnetic resonance imaging, DWI = diffusion-weighted imaging, ADC = apparent diffusion coefficient, FLAIR = fluid attenuated inversion recovery

Contrast-enhanced head MRI performed on hospital day 3 found that the high signals on DWI had disappeared. However, the FLAIR image showed ribbon-like high signals in the cerebral cortex, and no contrast effect was observed (Fig. 3). In subsequent additional tests, rheumatoid factor (RF) was 85 IU/L and anti-cyclic citrullinated peptide (CCP) antibody was elevated at 223.7 U/mL, while myeloperoxidase-anti-neutrophil cytoplasmic antibodies (MPO-ANCA) and proteinase-3-anti-neutrophil cytoplasmic antibodies (PR3-ANCA) were negative. Lumbar puncture showed a cell count of 68/μL (monocytes 14, polynuclear cells 54), protein 40 mg/dL, and glucose 52 mg/dL. Single photon emission computed tomography (SPECT) performed on day 6 showed decreased accumulation in the right temporoparietal region. Spinal fluid testing performed on day 10 showed an elevated interleukin-6 (IL-6) of 271 pg/mL, but anti-CCP antibody was normal at 3.7 U/mL. Because of the elevated cerebrospinal fluid cell count and the patient’s history of herpes encephalitis, she was temporarily treated with a continuous intravenous infusion of acyclovir 1500 mg/day until the polymerase chain reaction test was confirmed to be negative. The patient was finally diagnosed with RMSA. On day 16, continuous infusion of methylprednisolone 1000 mg daily was started for 3 days, and it was repeated on day 28. Her symptoms then gradually resolved, and the high signal on the FLAIR image also disappeared. The patient was discharged on day 44 with only a slight attention deficit on neurological examination.

## Discussion

The present patient was given a diagnosis of “definite RA” based on a score of 7 on the new 2010 American College of Rheumatology/European League Against Rheumatism (ACR/EULAR) classification criteria. Rheumatoid meningitis (RM) is defined as a condition of inflammation of the meninges or dura mater with cell infiltration. In the past, most RM cases were reported as autopsy cases, but the number of reported cases of RM has increased along with the increase in patients with long-standing disease and advances in diagnostic imaging. In many patients, RA is in the very early stage or long-term, and the disease duration is said to be ≥10 years for 50% or more [3]. The present patient presented 6 months after her diagnosis of RA, and thus was in the comparatively early stages of the disease; although the reason for that is not clear, it is consistent with earlier reports [4, 7]. In addition, although it did not correlate with her arthritic activity, the onset of RM was sudden and at a time when her RA activity was stable. There have been scattered reports of the use of intravenous steroid pulse therapy and oral steroids (1 mg/kg) [7, 8]. Even when there is improvement with treatment, caution must be taken regarding possible relapse or recurrence, but the present patient did not relapse up to 6 months later.

The neurological symptoms of RM may be hemiplegia, monoplegia, impaired consciousness, psychiatric symptoms, convulsions, and sensory impairment [9]. These symptoms are usually slowly progressive or transient [8]. However, to the best of our knowledge, there have been only two reports of patients with stroke-like attacks [10, 11]. Thus, although rare, RMSA should be included in the differential diagnosis of acute ischemic stroke presenting within the time window for thrombolytic therapy. The present patient is the first reported case of RMSA who was treated with rt-PA. MRI is helpful to differentiate it from acute ischemic stroke when it is performed on hospitalization.

The present patient’s symptoms rapidly improved in the early treatment period. It is possible that the brain-protective agent, edaravone, which was co-administered with intravenous rt-PA, contributed to that improvement. Free radicals have been reported to be involved in the vasculitis and cerebral edema associated with encephalitis as well [12], and to some extent, the administration of edaravone, a free radical scavenger, makes sense. MRI and biopsy findings have primary importance in the diagnosis of RM [3, 8]. A meningeal biopsy was not performed in the present case because of the rapid improvement of symptoms. However, the MRI findings were typical for RM, with a restricted ADC at the subarachnoid space adjacent to the right frontotemporal cortex. This linear high-intensity lesion on DWI is thought to result from proteinaceous debris accumulation at the subarachnoid space adjacent to the parenchyma with meningeal lymphocytic infiltration [13].

Evidence to date indicates that endovascular procedures provide clinical benefit in selected patients with acute ischemic stroke [14–17]. Thus, there is a trend toward CT-based prompt intravenous administration of rt-PA treatment rather than more time-consuming MRI study. In a comprehensive meta-analysis, stroke-mimicking patients were found to have a significantly lower risk for intracerebral hemorrhage and systemic complications than patients with true acute ischemic stroke (risk ratio = 0.33, 95% confidence interval, 0.14–0.77; *p* = 0.01) [18]. However, if the patients with RMSA would be susceptible to hemorrhagic transformation after receiving iv-tPA, it should be better to adopt the MRI first strategy for every patients with a history of RA. In the present case, it is particularly important that neither adverse events nor bleeding complications were observed, suggesting the safety of CT-based thrombolytic therapy even in RA patients.

## Abbreviations

ADC: Apparent diffusion coefficient
ASPECTS: The Alberta Stroke Program Early CT Score
CT: Computerized tomography
DWI: Diffusion weighted image
FLAIR: Fluid attenuated inversion recovery
MRI: Magnetic resonance imaging
NIHSS: National Institute of Health Stroke Scale
RA: Rheumatoid arthritis
RMSA: Rheumatoid meningitis presenting with a stroke-like attack
